# Perceptions of sarcopenia in patients, health and care professionals, and the public: a scoping review of studies from different countries

**DOI:** 10.1007/s41999-024-01132-5

**Published:** 2025-01-06

**Authors:** Emma Grace Lewis, Christopher Hurst, Linda Errington, Avan A. Sayer

**Affiliations:** 1 AGE Research Group, Translational and Clinical Research Institute, Faculty of Medical Sciences, Newcastle University, Newcastle upon Tyne, UK; 2 NIHR Newcastle Biomedical Research Centre, Newcastle upon Tyne Hospitals NHS Foundation Trust, Cumbria Northumberland Tyne and Wear NHS Foundation Trust and Faculty of Medical Sciences Newcastle University, Newcastle upon Tyne, UK; 3 School of Biomedical Nutritional and Sport Sciences, Newcastle University, Newcastle upon Tyne, UK

**Keywords:** Sarcopenia, Older people, Perceptions, Ageing, Qualitative research

## Abstract

**Rationale and objective:**

Perceptions of sarcopenia have rarely been explored, yet understanding these will be key for successful translation of sarcopenia research findings into meaningful benefits for patients and the public. This scoping review aimed to explore how sarcopenia is perceived amongst patients, health and care professionals (HCP), and the public in different countries.

**Methods:**

Seven electronic databases were searched from inception up to December 2023 with no geographical or language limitations. Studies were included if they were peer-reviewed research of any design where the focus related to perceptions of sarcopenia. Studies using alternative descriptors only, such as “skeletal muscle weakness/loss” were excluded. Study characteristics were charted, and thematic synthesis conducted.

**Results:**

Following independent screening of 11,533 records, 20 articles were included in this review representing 19 countries. Five studies focused on patient perceptions, 11 investigated HCP, and four the public. Three key themes were identified: (1) Low awareness of sarcopenia—among all groups, its nature as a disease was contested. (2) The “know-do” gap in healthcare—even where knowledge among HCP existed, this had not translated into clinical practice, in part, due to perceptions of sarcopenia and its management. (3) Experiencing weakness—living with sarcopenia had physical and psychological sequalae.

**Key conclusions:**

These findings reveal perceptions that may be contributing to the slow adoption of sarcopenia prevention, screening, diagnosis, and management. Addressing these areas has the potential to aid translation of sarcopenia research findings into improved clinical care and benefits for patients and the public.

**Supplementary Information:**

The online version contains supplementary material available at 10.1007/s41999-024-01132-5.

## Key summary points

**Aim:**

This scoping review aimed to explore how sarcopenia is perceived amongst patients, health and care professionals (HCP), and the public, in different countries.

**Findings:**

Sarcopenia is poorly understood by patients, HCP and the public, and its nature as a “disease” is contested. There is a “know-do” gap in diagnosis and management of sarcopenia. Living with sarcopenia has both physical and psychological impacts, but patient perceptions of sarcopenia are under-investigated, particularly for men, patients from under-served groups, and those living with multiple long-term conditions (MLTC).

**Message:**

A better understanding of sarcopenia perceptions across a range of geographical settings and populations highlights areas that could be addressed to improve clinical care and benefits for patients and the public.

**Supplementary Information:**

The online version contains supplementary material available at 10.1007/s41999-024-01132-5.

## Introduction

Sarcopenia can be defined as the accelerated loss of muscle strength and mass, commonly but not uniquely associated with ageing [1]. The prevalence of sarcopenia in older community-dwelling adults is estimated at between 9.9 and 40.4%, but depends on the diagnostic criteria used, and the setting applied [2].

People with sarcopenia have an increased risk of mortality and a host of other adverse outcomes such as falls, functional dependency, hospital admission and a reduced quality of life [3, 4]. A recent systematic review of the relative costs of sarcopenia found that healthcare costs were significantly higher for those with sarcopenia compared with those without [5]. Opportunities have been identified in taking a lifecourse approach to sarcopenia prevention, with an emphasis on optimising peak muscle mass and strength in adulthood [6].

There has been a rapid increase in research in recent years, for example establishing diagnostic criteria [7], developing screening tools [8], exploring potential pharmacological treatments [9], and investigating resistance exercise-based interventions [10], yet understanding perceptions of sarcopenia is key in order to translate this research into patient and public benefit. The knowledge, attitudes and practice of health and care professionals (HCP) in relation to sarcopenia have been described [11], concluding that limited knowledge was hindering clinical practice. Patient and HCP perceptions are key to driving improved quality of care, for example they have been investigated to better understand older adults’ self-management of long-term conditions, as well as experiences of emergency care for care home residents [12, 13]. Perceptions of patients and the public are also likely to be integral to developing effective approaches to diagnosis, treatment, and prevention of sarcopenia, and will allow innovations and service developments to be measured against older people’s valued capabilities.

Most studies in this field have taken a single country or regional scope, yet global variations in grip strength suggest a need to address sarcopenia as a global public health challenge [14]. Therefore, this scoping review takes an international perspective to investigating perceptions of sarcopenia by including research in any language and any population.

As such, the aim of this scoping review was to explore how sarcopenia is perceived among patients, HCP, and the public in different countries. To achieve this through a thematic synthesis of peer reviewed research investigating sarcopenia perceptions published to date.

## Methods

Arksey and O’Malley’s framework [15] with enhancements by Levac et al. [16] was used to conduct the scoping review. A scoping review was most appropriate as it allowed for an exploratory overview of the current knowledge base of sarcopenia perceptions and aligned with the objective of identifying gaps in the literature [17]. The Preferred Reporting Items for Systematic reviews and Meta-Analyses extension for Scoping Reviews (PRISMAScR) checklist was followed for comprehensive, transparent, and coherent reporting of the literature [17]. The review protocol was registered on Open Science Framework on the 9th November 2023 10.17605/OSF.IO/R3KXJ.

Key search terms and inclusion criteria were established through collaborative discussion between co-authors, including a medical librarian with research methodology expertise. This ensured an effective search strategy which would appropriately address the review question.

Articles were deemed suitable for inclusion if they were peer-reviewed primary or secondary research of any design (qualitative, quantitative or mixed-methods) where the focus related to perceptions of sarcopenia in any population. The setting, language, date of publication and geographical area were not restricted. In order to meet our scoping review objectives, the term “sarcopenia” must have been used, and poorly defined conditions such as “poor muscle quality” “skeletal muscle weakness/loss” were excluded.

### Search strategy

The databases MEDLINE, CINAHL, EMBASE, PsycINFO, Scopus, Web of science, ProQuest social science were searched by a medical librarian with research methodology expertise between 1st November and 1st December 2023. Key search terms were truncated, and wildcards used and adjusted in line with the respective requirements of each database to maximise results. Search terms were connected via several combinations using the boolean operators AND or OR as appropriate. All databases were searched from inception with no date limitations. Final search terms included: Sarcopenia OR sarcopeni* AND qualitative research OR grounded theory OR qualitative OR mixed method* OR interview* OR ethnograph* OR phenomenol* OR observation* OR focus group* OR interpretive phenomenological analysis OR narrative OR thematic analysis AND perception OR perceive OR belief* OR perspective* knowledge OR understand* OR attitude* OR recogni* OR aware* OR believe OR view* OR opinion* OR experience* OR reflect* OR concept* OR representation*. Table 1 shows the full search strategy for MEDLINE.

**Table 1:** The full search strategy for Ovid MEDLINE(R) database

Database: Ovid MEDLINE(R)
**Search Strategy:**
**1** Sarcopenia/
**2** sarcopeni*.ti,ab,kf
**3** 1 or 2
**4** qualitative research/
**5** grounded theory/
**6** (qualitative or mixed method* or interview* or grounded theory or ethnograph* or phenomenol* or observation* or focus group* or narrative or thematic analysis).ti,ab,kf
**7** (perception or perceive* or belief* or perspective* or knowledg* or understand* or attitude* or recogni* or aware* or believe or view* or opinion* or experience* or reflect* or concept* or representation*).ti,ab,kf
**8** 4 or 5 or 6 or 7
**9** 3 and 8

### Study selection

All search results were exported into EndNote 21 for de-duplication. References were then exported to the mobile and web application, Rayyan for screening of titles and abstracts [18]. Two blinded reviewers (EGL and CH) screened titles and abstracts for eligibility and relevance and rated each title (I = Include, E = Exclude, U = undecided). Full texts of eligible abstracts were reviewed by the same two independent reviewers to confirm eligibility. The two reviewers met to discuss any conflicts of decision after screening of titles and abstracts, and after reviewing the full texts and a consensus was met. Forward and backward citation tracking was performed for the papers selected and any additional papers identified for inclusion were decided by consensus by EGL and CH.

### Data extraction and analysis

A data-charting form was created by the first author in Microsoft Excel. This included the first author and their affiliations, the title of the paper, country, year published, journal, funding and declared conflicts of interest, the study’s stated aim, design, data collection method, description of the sample and a summary of the main findings. The study characteristics were summarised in a table, by groupings of patients, HCP, and the public (Table 2). The first author noted their initial impressions and reflections which would contribute to the generation of initial codes. Thematic synthesis was conducted, using integration via assimilation of evidence types to organise and describe the data in rich detail [19]. As this was a scoping review, a quality assessment of articles was not conducted, and critiques were noted only where relevant to the topic of sarcopenia perceptions.

**Table 2:** Summary characteristics of the included papers

Authors	Country	Funding	Aim	Design and data collection method	Sample
Patients					
Rush et al. (2011) [24]	Canada	Public	To understand the meaning of weakness for older adults’ and their perceptions of its association with ageing	Qualitative interviews	13 older community-dwelling adults
Evans et al. (2011) [23]	USA	Private biopharmaceutical company	To develop a patient-reported outcome (PRO) to assess reduced muscle strength in sarcopenia	Qualitative interviews	12 older adults
Beaudart et al. (2020) [21]	Belgium, Spain	Public–private partnership	To identify critical outcomes for sarcopenia and to select the 5 most important outcomes that will be used in a further discrete-choice experiment (DCE)	Literature review, expert panel and three focus group discussions	19 older adults
Hiligsmann et al. (2020) [22]	Belgium, France, Germany, Italy, Spain, and Switzerland	Public–private partnership	To evaluate patient's priorities for sarcopenia outcomes (from five pre-determined outcomes decided by prior work by Beaudart et al. 2020)	Cross-sectional survey	216 older adults diagnosed with sarcopenia by EWGSOP, FNIH or IWGS*
Zanker et al. (2022) [25]	Australia and New Zealand	Public and Public–private partnership	To develop guidelines, informed by healthcare consumer values and preferences, for sarcopenia prevention, assessment, and management for use by clinicians and researchers in Australia and New Zealand	Three-phase modified Consumer Expert Delphi study	24 older adults
Health and Care Professionals					
Yaxley et al. (2011) [38]	Australia	Public	To determine whether dietitians understand and use the terms starvation, sarcopenia, and cachexia and provide targeted treatment strategies accordingly	Cross-sectional online survey	221 members of the Dieticians Association of Australia
ter Beek (2016) [37]	Belgium, the Netherlands Norway and Sweden	None, no conflicts of interests	To determine whether dietitians in selected European countries have ‘sufficient knowledge’ of malnutrition, starvation, cachexia and sarcopenia, and use these terms in their daily clinical work	Cross-sectional online survey	369 respondents
Reijnierse et al. (2017) [26]	the Netherlands	Public	To assess knowledge and practice of sarcopenia diagnosis and management among Dutch healthcare professionals	Longitudinal survey questionnaire	223 HCP
Nakahara et al. (2018) [29]	Japan	None, no conflicts of interest	To evaluate and compare the relative use of sarcopenia and cachexia evaluations among dietitians and associated healthcare professionals in a diverse range of settings	Cross-sectional online survey	683 HCP
Offord et al. (2019) [40]	The United Kingdom	None, conflicts of interest	To survey UK healthcare professionals to understand how sarcopenia and frailty are diagnosed and managed in current UK practice	Cross-sectional online survey	61 HCP
Kiss et al. (2020) [39]	Australia	None, no conflicts of interest	To determine the awareness, perceptions, and practices of Australian oncology clinicians regarding malnutrition and sarcopenia in people with cancer	Cross-sectional online survey	111 HCP
Silva et al. (2020) [31]	Brazil	Not mentioned	To describe the knowledge and practices of primary care nurses on sarcopenia screening	Qualitative interviews	24 nurses
Yeung et al. (2020) [27]	Australia and New Zealand	Public	To describe the current knowledge and practice of sarcopenia diagnosis and treatment among health‐care professionals before, directly after and 6 months after a professional development event on sarcopenia	Longitudinal survey questionnaires	250 HCP
Guralnik et al. (2022) [30]	USA	Public–private partnership	To evaluate US physicians' familiarity with sarcopenia and its use in their practices	Qualitative interviews and cross-sectional online survey	9 physicians interviewed, 253 physician respondents
Lu et al. (2023) [28]	China	Public	To analyse and compare the knowledge, attitude, and practice regarding sarcopenia between orthopaedics and geriatrics professionals	Cross-sectional online survey	220 HCP
Verstraeten et al. (2023) [32]	the Netherlands	Private and public–private partnership	To assess sarcopenia awareness and knowledge; perception of responsibility; current screening, diagnosis, and treatment practices; and barriers and enablers to screening/diagnosis and treatment of sarcopenia among geriatric rehabilitation health care professionals in the Netherlands. Adequate knowledge was assessed against EWGSOP/EWGSOP2 guidelines	Cross-sectional online survey	501 HCP
The public					
Van Ancum et al. (2020) [33]	the Netherlands	Public	To describe the current knowledge about sarcopenia in a cohort of community-dwelling adults attending health educational events. And to correlate self-perceived muscle health with objective muscle measures	Cross-sectional survey, and physical measures including bioimpedance analysis, hand-grip strength and 4 m walk speed test	197 older adults
Gilliot et al. (2021) [36]	Belgium	None, no conflicts of interest	To investigate with Google Trends whether the clinical importance of sarcopenia is reflected in public interest in the disease. Sarcopenia was compared with search trends on the topics of "dementia" "polypharmacy" "osteoporosis" and "frailty"	Analysis of search tool “Google trends”	Google trends was queried for data from Jan 2004 to Jan 2020 with geographical location not limited (worldwide)
Keng et al. (2023) [35]	Malaysia	None, no conflicts of interest	To investigate "knowledge of sarcopenia" among the public in Malaysia	Cross sectional online survey recruited via social media platforms	202 respondents
Lee, Shu-Chun et al. (2023) [34]	Taiwan	None, no conflicts of interest	To evaluate the level of awareness of sarcopenia among older adults and to develop and test the reliability and validity of the Sarcopenia Knowledge Questionnaire	Cross-sectional paper survey of participants recruited through hospital outpatient department	293 older adults

* European Working Group on Sarcopenia in Older People (EWGSOP), Foundation for the National Institutes for Health (FNIH), or International Working Group on Sarcopenia (IWGS)

The original articles were uploaded in full as data files to Nvivo 14 and the results and discussion sections were each coded by the first author according to Braun and Clarke’s thematic analysis framework [20] to develop key themes and narratively report the findings (Table 3). Themes were discussed in a meeting with all co-authors at the point of reviewing and refining the key themes. The first author is an academic geriatrician working in an aging research group with expertise in sarcopenia. Reflexively, this background means the first author is close to the research topic and identified with many of the HCP survey findings.

**Table 3:** Describing the themes with illustrative quotations

Theme title	Summary of the idea/concept	Illustrative quotation	Papers and references
Low awareness of “sarcopenia”	There is a globally poor recognition, understanding and use of the term sarcopenia	“Most likely, the awareness for sarcopenia among laymen is insufficient or even non-existing”. [36]	13 papers, 36 references
The contested nature of sarcopenia- a disease or normalised	Among all groups (patients, the public and HCPs) there was ambiguity over the degree to which sarcopenia was or should be medicalised vs normalised as part of the ageing process	“Nearly all health care professionals correctly identified sarcopenia as the occurrence of low muscle strength, muscle mass, and function; however, it was not often identified as a disease.” [32]	6 papers, 11 references
The “know-do” gap in healthcare	Sarcopenia knowledge was repeatedly shown not to translate into clinical practice across a range of HCPs and settings	“A ‘know-do’ gap exists in the management of frailty and sarcopenia. For both conditions, exercise training is known to be effective, and yet patients with frailty or sarcopenia are not being offered such programmes.” [40]	9 papers, 26 references
Not identifying sarcopenia	One of the ways in which the “Know-do” gap manifested is that HCPs did not take actions to screen or diagnose sarcopenia	“The finding that respondents reported limited use of the terms cachexia (30%), sarcopenia (12%) and starvation (3%) in their daily work documentation might indicate that these nutrition-related disorders are suboptimally recognized in clinical practice.” [37]	9 papers, 17 references
Not diagnosing sarcopenia correctly	Published guidelines were applied not widely applied, and malnutrition definitions or frailty tools were sometimes used	“Of the health‐care professionals reporting the use of muscle mass as a diagnostic criterion at follow‐up, more than half used inappropriate methods such as calf circumference and skinfold thickness.”[27]	7 papers, 15 references
Whose responsibility is sarcopenia?	One focus of enquiry among the survey studies of HCPs was attempting to answer the question of which cadre and specialism should take the primary responsibility for the work of screening, diagnosis and management. It was argued that the lack of clarity on this was contributing to the “know-do” gap	“Dietitians perceived themselves as responsible for diagnosing sarcopenia, but the responsibility of dietitians was underrecognized by other professions and physicians and PTs/OTs pointed toward each other in terms of who is responsible for diagnosis. This finding shows a lack of agreement with regard to whose responsibility it is to diagnose sarcopenia in geriatric rehabilitation.”[32]	7 papers, 11 references
Why diagnose sarcopenia?	In addition to a focus on technical and system-related factors, one survey asked respondents what would motivate them to screen for sarcopenia. Another paper asked survey respondents for their reasons for assessing frailty, yet the “why” of sarcopenia diagnosis was notably missing from most papers	“A majority of physicians would be motivated to screen for sarcopenia out of concerns for fall and injury prevention (60%) and for the loss of ability to remain independent and mobile (53%).” [30]	2 papers, 2 references
Clinical scepticism towards exercise and nutrition	There was a sense that “lifestyle” measures would be unlikely to be adopted successfully, and that prevention and treatment were blurred when it comes to sarcopenia	“I can screen for it [sarcopenia], detect it, and diagnose it. But if there’s no one to do the actual meal part for them — the groceries, the cooking — then that’s it. As a provider, I can’t help them.” — Geriatric Medicine Interviewee [25]	3 papers, 5 references
Positive attitudes towards resistance exercise	In contrast with HCPs, patients and the public generally expressed enthusiasm for the idea of exercise and dietary prescriptions for prevention and treatment of sarcopenia and viewed medication less enthusiastically	“Most consumers identified willingness to be involved in research for both exercise (n = 21, 88%) and dietary (n = 15, 62%) studies. However, only one- third (n = 8, 33%) of consumers expressed willingness to be involved in clinical trials of medication for sarcopenia.” [24]	4 papers, 10 references
Comparisons with frailty	Frailty was often mentioned in relation to and alongside sarcopenia, revealing that among some HCPs and patients there is a perception of overlap or closeness between the two concepts	“Negative responses to weakness and ageing reflect other findings of resistance by older adults to being portrayed as frail, sick, dependent, and in need of health services (Chater, 2002; Grenier & Hanley, 2007).” [26]	6 papers, 7 references
System factors as “barriers”	Papers reporting surveys of HCPs found that several system-related factors hindered the implementation of guidelines. Examples of system factors included a lack of equipment for making a diagnosis and poor recognition and reimbursement by insurance companies	“The acquisition of a device to measure muscle mass was one of the most reported bottlenecks. Clearly, financial aspects such as the acquisition of even a relatively cheap bioelectrical impedance analysis (BIA) device, creates huge barriers for implementation.” [26]	5 papers, 9 themes
Experiencing weakness	The experience of muscle weakness in the context of ageing was usually found to have both physical and emotional components. The subjective experiences of weakness did not correlate with objective measures in one study	“The meaning of weakness for study participants was twofold: physically as loss of functional ability and emotionally as emerging passivity in character and personhood. Rather than subscribing to the stereotypical image of older adults as weak, participants in the current study perceived themselves as ‘a strong person’” [24]	3 papers, 15 references
Sarcopenia is validated by its adverse outcomes	The importance of sarcopenia diagnosis and management is justified and understood with reference to its impact on older people and the adverse outcomes associated with it	“Most subjects (n = 10) reported experiencing emotional impacts because of lack of muscle strength. Eight subjects reported feelings of frustration.” [2]	9 papers, 16 references

## Results

**Fig. 1:**
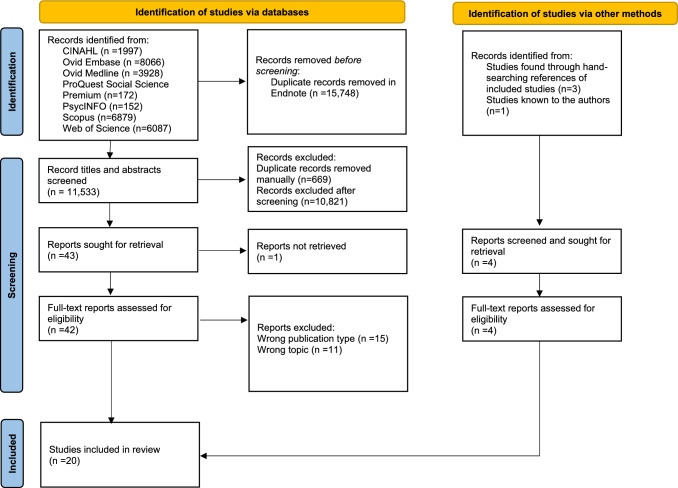
PRISMA flow diagram

In total, 27,281 records were identified through database searching. Following duplicate removal, 11,533studies were screened by title and abstract. 42 full-text articles were retrieved and screened for eligibility – conference abstracts and publications on topics other than that of this scoping review, were excluded. In total, twenty studies were included in this review. The flow of articles through identification to final inclusion is shown in the PRISMA diagram Fig. 1.

### Study characteristics

Twenty studies were included in the analysis, from 19 countries representing all continents except Africa. The majority derived from European countries, the most common being Belgium and the Netherlands. Studies were published between 2011 and 2023, the median, six, were published in 2020 (Table 2).

### Studies investigating patient perceptions

Five studies, including a total of 284 participants, sought to investigate “patients” or people living with sarcopenia. Two papers described components of the same project, that aimed to identify five critical outcomes of sarcopenia [21], that were used to perform a discrete choice experiment to evaluate patients’ priorities for sarcopenia outcomes [22]. Two papers described how their participants were selected, thus defining a “patient”. Evans et al. used measures of physical performance, such as low gait speed and a score of between 4 and 9 on the short physical performance battery and excluded any diagnoses that may lead to secondary sarcopenia [23]. One study used recognised diagnostic guidelines for sarcopenia to define the included patients [22]. The others permitted participants to self-identify as “living with weakness” [24], or as people “living with sarcopenia” [25]. Two studies acknowledged funding from Amgen, an independent multinational biotechnology company [23, 25]. Other funders included non-profit organisations representing the dairy industry [25] and a non-profit organisation termed the European Society for Clinical and Economic Aspects of Osteoporosis, Osteoarthritis and Musculoskeletal Diseases (ESCEO) [21, 22]. All studies included a majority of “Caucasian”, female participants and only 6 from 13 of the participants of Rush et al.’s study had multiple long-term conditions (MLTC) [24]. These study characteristics are described in detail in Appendix 1.

### Studies investigating the perceptions of Health and Care Professionals

Eleven studies aimed to investigate the perceptions of HCP in relation to sarcopenia. In total, 2925 HCP participants were included. Two studies used similar methods in evaluating the impact of a professional training event termed “the Sarcopenia Roadshow” with follow-up surveys conducted months later to investigate change in practice and assess knowledge attrition [26, 27]. The other studies were cross-sectional, most using online surveys, analysed quantitatively, to compare knowledge and attitudes between specialities [28], and across healthcare settings or cadres [29]. Two studies employed qualitative interviews with HCP [30, 31]. Work was funded by both public funding such as the Medical Science and Technology Project of Zhejiang Province [28] and through public–private partnerships e.g. Danone Nutricia Research [32] and the Alliance for Aging Research (funded by Abbott Nutrition, Biophytis, Cytokinetics, Metabolic Technologies, LLC, Nestle Health Science, and Pfizer Inc.) [30].

### Studies investigating public perceptions of sarcopenia

Four publications aimed to investigate knowledge or awareness of sarcopenia among the public. Three from four were un-funded, whereas The European Union Horizon 2020 research and innovation programme provided a grant for the Van Ancum et al. study which incorporated survey data with physical measures such as hand-grip strength [33]. The other studies were cross-sectional survey questionnaires [34, 35], and an analysis of the search tool “Google trends” [36]. The survey responses represented 692 participants, these were a majority female, highly educated, and physically active. Despite recruiting participants from a hospital outpatient department, Lee et al. reported only 27 (9%) of survey respondents had > 3 comorbidities and 215 (73.4%) exercised either daily or more than once weekly [34].

### Results of the thematic analysis

**Fig. 2:**
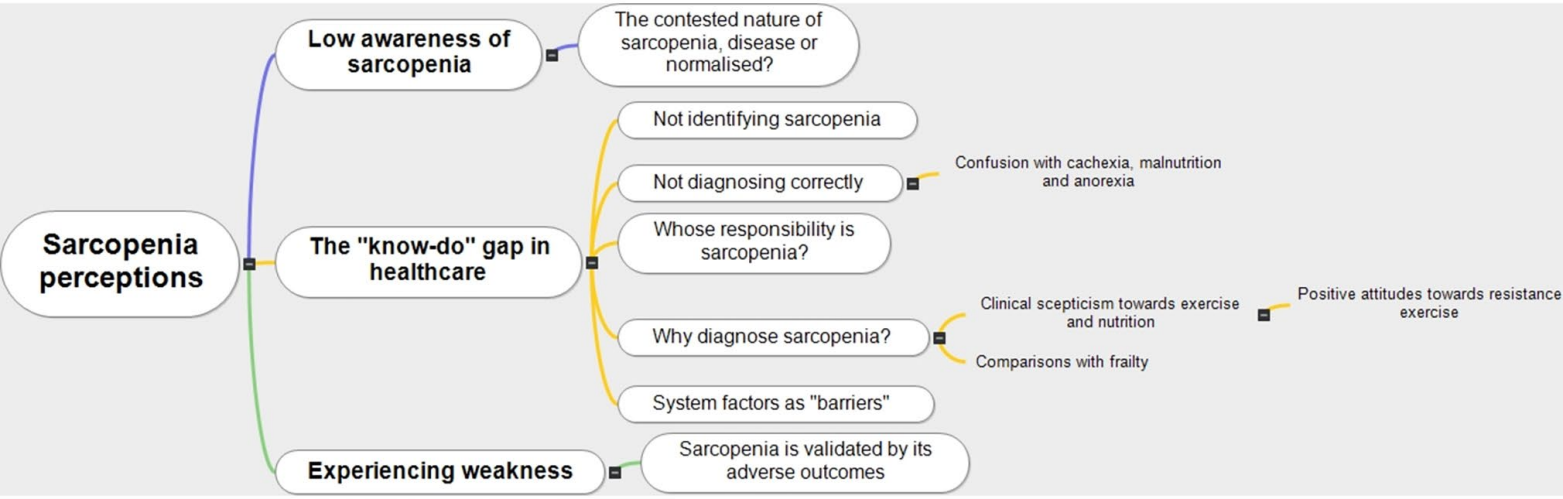
Diagram illustrating the key themes and sub-themes

Table 2 outlines the title and concept of each theme and provides an illustrative quotation. Figure 2 depicts the three key themes and their sub-themes.

### Key theme 1. Low awareness of sarcopenia

All groups demonstrated a limited knowledge and/or awareness of sarcopenia. For the purposes of this review, “knowledge” refers to facts, information and skills relating to the subject of sarcopenia, while “awareness” relates to knowledge or perception of the existence of sarcopenia. In Evans et al.’s study, that aimed to develop a patient reported outcome measure, the authors determined that awareness of sarcopenia was so low that each questionnaire item should be prefaced by *“considering your loss of muscle strength”*, given that *“most individuals are not aware of the existence of sarcopenia”* [23]. A study comparing the relative search volumes of several important geriatric conditions evidenced the low public awareness of sarcopenia. Sarcopenia had the second lowest search volume- the lowest was polypharmacy-, whereas dementia, osteoporosis and frailty were the three most commonly searched terms [36].

Where sarcopenia *was* recognised, it was sometimes not perceived as “a disease” as compared with a syndrome, a condition, or a normalised part of ageing. This ambiguity was found across patients, HCP and the public. A survey of Chinese HCP found sarcopenia was infrequently classified as a disease by geriatricians (n = 12/44, 22.7%) and orthopaedic surgeons (n = 73/176, 41.5%) [28]. Similarly, a survey of HCP working in geriatric rehabilitation found that while 62.1% (n = 296/477) of participants recognised that sarcopenia consisted of low muscle mass, strength, and physical performance, only 10% (n = 50/487) recognised sarcopenia as “a disease” [32]. Patients interviewed about their perceptions of muscle weakness were ambivalent and contradictory in their attitudes, but to some extent did attribute weakness to normal ageing [24]. The authors of a survey of the Malaysian public concluded that participants viewed sarcopenia as *“a natural component of the aging process”*, rather than a preventable problem [35].

### Key theme 2. The “know-do” gap in healthcare

The following themes focused on the most-studied, and surveyed group- HCP. A recurrent finding in the papers that surveyed HCP was that even where knowledge and understanding existed, this did not translate into clinical practice. Two very similar studies [26, 27], found that despite a majority of HCP having the intention to diagnose sarcopenia, very few had incorporated sarcopenia screening, diagnosis and management into their practice at the evaluation point at around six months post a professional training event [26, 27].

Part of this problem was due to a lack of adherence to sarcopenia guidelines. Yeung et al. found that sarcopenia diagnoses were being made based on inappropriate definitions (such as the European Society for Parenteral and Enteral Nutrition malnutrition definition, frailty by Fried, and frailty by Rockwood) [27]. There also appeared to be confusion between diagnostic criteria for cachexia, starvation, malnutrition, and sarcopenia among dieticians [37, 38]. HCP did not screen for sarcopenia in their patient groups due to competing clinical priorities. A study in primary care in Brazil found that nurses felt screening for, and preventing other conditions, such as diabetes and hypertension was a higher priority [31].

To explain the low adherence to guidelines, the survey studies of HCP enquired about perceived “barriers”, often attributing the “know-do” gap to technical and systemic factors. The study by Reijnierse et al. entitled “*Lack of knowledge and availability of diagnostic equipment could hinder the diagnosis of sarcopenia and its management”* exemplifies this practice [26]. The lack of recognition of sarcopenia by healthcare financing systems was raised as a contributor to the “know-do” gap. Sarcopenia was either not recognised by national treatment code lists, thus not permitting the reimbursement of healthcare costs [32], or was not recognised by health insurance companies [26, 30]. Additional practical concerns were raised, such as a lack of access to tools, diagnostic equipment, and of time constraints [26, 27, 30, 32]. Many of these papers grappled with the challenge of whose responsibility it should be to screen for, diagnose, and manage sarcopenia. The perceptions of responsibility for diagnosing sarcopenia among HCP treating cancer patients varied, yet *“93% agreed this was a component of their role”* [39]. In contrast, 52% of nurses surveyed in another study felt it was not their role to diagnose sarcopenia [27]. When a consumer group was surveyed on this question, (n = 11) 46% responded that they had no preference for which professional group diagnosed sarcopenia [25].

Despite a general recognition of the adverse outcomes associated with sarcopenia, there remained uncertainty over which patients will benefit from a diagnosis. “*A majority of physicians would be motivated to screen for sarcopenia out of concerns for fall and injury prevention (60%) and for the loss of ability to remain independent and mobile (53%).*” [30]. While survey respondents could list reasons for assessing frailty, which included “*prognostic purposes, to aid decision-making, and because it was recommended by guidelines*” the authors, notably, did not ask the same question as part of their sarcopenia survey [40]. US physicians expressed a scepticism around the potential for diet and exercise as a preventative measure and treatment for sarcopenia because of negative assumptions about their patients’ attitudes toward these interventions. “*When asked to roughly estimate how many of their patients would suffice with diet and exercise as the only interventions to address sarcopenia, responses centred around the middle: 35% saying “about half” and 38% saying “a few or some.”*” [30]. Illustrative quotes from the qualitative component of this study demonstrated a sense of frustration or futility in offering exercise or dietary measures “*For many older individuals, exercise isn’t something they relish. You have to disguise it in other things to get them to engage. Even getting up and getting out is a form of physical activity.*” — Physical Medicine & Rehabilitation Interviewee [30].

In contrast, the older adults questioned about their attitudes to treatments and prevention were largely positive, with 71% of participants willing to increase both their protein intake and physical activity levels to prevent sarcopenia, and 76% reporting they would be motivated to undergo muscle strength training and a high protein diet if diagnosed with sarcopenia [33]. Zanker et al. similarly found their “consumer” group of interested healthcare users “*mostly preferred to undertake resistance exercise (n* = *18, 75%) followed by taking prescription medications (if available; n* = *17, 71%) and making dietary modifications (n* = *16, 67%)”* for the prevention of sarcopenia.

The conceptual closeness between sarcopenia and frailty was either directly or obliquely discussed in several papers. Older people living with muscle weakness described negative connotations associated with sarcopenia, that was likened to the stigma attached to frailty [24]. The topic arose in qualitative interviews with HCP; “*When we’re writing papers and teaching, we’ll say ‘sarcopenia,’ but when we’re talking in clinical care, we’ll say ‘frailty.’*” — Geriatric Medicine Interviewee [30]. This perception of the closeness between the two concepts also influenced clinical practice, with frailty criteria used as a method of diagnosing sarcopenia [27, 32].

### Key theme 3. Experiencing weakness

Few studies investigated the lived experience of sarcopenia, but a common finding across these studies was that living with sarcopenia has both physical and psychological aspects. The qualitative study conducted in Canada found older people conceptualised their (muscle) weakness as both an “inability”, and as “turning inward” [24]. Participants took part in several techniques for resisting weakness, through techniques and behaviours such as “motivating self-talk”, “keeping busy and active” and “validating as a strong person” [24]. Participants focused on their physical functioning when discussing their weakness as “inability”, but the concept of “validating as a strong person” reflected their psychological resilience to cope with losses [24]. Evans et al. found that people living with sarcopenia experienced both physical and psychological impacts of their muscle weakness, including the troubling symptom of fatigue [23].

The importance of the adverse outcomes of sarcopenia was widely recognised, and used to justify and validate the call for improved diagnosis and management. Work aiming to identify critical outcomes of sarcopenia, through literature review, patient involvement and expert opinion identified five key outcomes “mobility (30%), followed by the ability to manage domestic activities (22%), the risk of falls (18%), fatigue (17%), and quality of life (14%)” [22]. In a survey of 197 older community-dwelling adults, despite a low level of knowledge and awareness of sarcopenia, important outcomes (falls, fractures, and admission to a nursing home) were correctly identified by most [33]. HCP recognised the importance of sarcopenia outcomes, but at different rates, with HCP working in orthopaedics less able to recognise cognitive impairment, increased mortality, or an increased economic burden as negative outcomes of sarcopenia [28].

## Discussion

This scoping review has synthesised the current peer-reviewed research to date investigating sarcopenia perceptions. Most of the included publications derived from European and North American countries (one from the United Kingdom). Only two upper middle-income countries were represented (Malaysia and Brazil, according to World Bank classifications) and none from Africa. This is an important gap in the literature, especially given low and middle-income countries have been shown to have lower normative hand-grip strength values and may have more to gain from public health interventions [14]. The characteristics of funding for these studies also revealed an important imbalance. Private companies and public–private partnerships invested in research with patients and HCP, whereas studies focusing on the public often received no clear source of funding. The included studies are all published within the last twelve years despite the fact that sarcopenia was first recognised in the 1980s [41]. This observation is likely to reflect the progress that has been made in sarcopenia’s translational journey. That more recent research has focussed on understanding how the concept is understood, with the most studied group being HCP, is an indication that researchers see the importance of perceptions of sarcopenia, particularly in the healthcare context, for sarcopenia to lead to meaningful patient benefit.

Knowledge and awareness of sarcopenia is low, particularly among the public. This finding has important implications for public health. Public participants in these studies have tended to be functionally independent, physically active, and educated. In one study, one third were retired HCP [33]. We can draw from this that broader public knowledge and awareness is likely to be lower than demonstrated, given that poorer health literacy is associated with lower educational attainment and disability [42]. Participation in research already suggests a level of engagement with health-related behaviours [43], which may explain some of the strongly positive attitudes expressed toward the prescription of resistance exercise and nutritional interventions. While welcome, this finding is likely to be explained by a “healthy volunteer” selection bias [43]. Nevertheless, this scoping review highlights that more should be done to publicise public health recommendations and the potential for sarcopenia prevention through resistance exercise and diet [44, 45].

The perception that sarcopenia was an inevitable and normalised aspect of ageing was surprisingly prevalent, even among HCP who greatly over-estimated the prevalence of sarcopenia in older age [30]. The belief that sarcopenia was a natural and irreversible part of ageing was the most prevalent reason given by US physicians for why their patients failed to address their loss of muscle mass and strength [30]. This finding echoes a scoping review investigating public perceptions of frailty in the UK where the authors similarly found that frailty is seen as part of the ageing process [46]. This may be partly explained by the conceptual closeness between the two, a sub-theme of this analysis. This perception of sarcopenia is in contrast with the Global Leadership Initiative in Sarcopenia (GLIS) Delphi study that aimed to establish expert consensus, conceptualising sarcopenia as a “generalised disease of the skeletal muscle” [47]. While the condition was understood to be important by all groups, through its association with important adverse outcomes such as falls and institutionalisation, this synthesis raises an ongoing ambiguity that exists over sarcopenia’s status as a “disease”, despite its receipt of an ICD-10 diagnostic code in 2016 [48].

Consistent with the previous review of HCP perceptions [11], the finding of low knowledge, and the gulf between knowledge and practice were both key themes. The authors of these studies tended to approach the problem of the “know-do gap” from a task and process-focussed lens. Enquiring about “barriers” and, for example, concluding that sarcopenia diagnoses are not being made due to a lack of diagnostic equipment [26]. This drew attention to the notable lack of discussion about the “why” and “when” of sarcopenia diagnosis and management, (as opposed to “how” and “who” questions). The perception that sarcopenia is conceptually close to and related to frailty, emphasises this disparity. In contrast with sarcopenia, frailty screening has been widely integrated into primary and secondary care services across the National Health Service, through the implementation of screening tools such as the electronic frailty index (eFI) [49]. The question of the clinical purpose of frailty screening has been addressed [50], and was reflected in HCP responses in the Offord et al. paper [40]. This analysis suggests that HCP need to be similarly persuaded of the potential benefit of a sarcopenia diagnosis for their patients, for the technical implementation “barriers” to be overcome. Clinical research in this area should prioritise investigating clinically relevant outcomes e.g. reduced falls-associated hospital admissions for patients with sarcopenia, in addition to benchmarking exercises to set best practice in delivery of therapies and services [51], to motivate HCP and lead to lasting application in clinical practice.

Patient perceptions of sarcopenia are under-investigated, particularly for men, patients with lower educational attainment, lower socio-economic groups, and those living with MLTC (See Appendix 1 for details). People with cognitive impairment have also been excluded by these studies necessitating consent and most requiring completion of a survey. This is an important gap given that sarcopenia is highly prevalent among people with dementia [52]. Where patients have been surveyed or interviewed, there has been a challenge in identifying the sarcopenia patient group, with only one study reporting the use of recognised diagnostic guidelines in their inclusion criteria. Evans et al. deliberately excluded those with conditions that may cause secondary sarcopenia [23], yet this may have disregarded and important group of patients with MLTC and sarcopenia. Analysis of UK Biobank data has shown that adults aged 40–70 years with MLTC had nearly twice the odds of probable sarcopenia, with authors concluding that this patient group may benefit from sarcopenia screening in mid-life [53]. The reliance on self-identification in these papers is also a significant weakness, particularly given the finding by Van Ancum et al. that the public both over- and under-estimated their muscle health [33]. This scoping review has highlighted an important gap in our understanding of the patient experience of sarcopenia, particularly for under-served groups.

### Strengths and limitations

This thematic synthesis has richly described the field of research investigating sarcopenia perceptions across a range of geographical settings and populations. Most of the survey questionnaires were devised by the individual study authors and were not replicated across settings, limiting comparability and any quantitative analysis in this scoping review.

## Conclusion and implications

These findings reveal perceptions that may be influencing the slow adoption of sarcopenia prevention, screening, diagnosis, and management. Addressing these areas has the potential to aid translation of sarcopenia research findings into improved clinical care and lead to meaningful benefits for patients and the public.

## Supplementary Information

Below is the link to the electronic supplementary material.

Supplementary file1 (DOCX 30 KB)

Supplementary file2 (DOCX 83 KB)

## Data Availability

Data will be made available on request.
