# Supplementary material for: Perceptions of sarcopenia in patients, health and care professionals, and the public: a scoping review of studies from different countries
Source: Eur Geriatr Med. 2025 Jan 6;16(1):99–113. doi: 10.1007/s41999-024-01132-5 (PMC11850425; doi:10.1007/s41999-024-01132-5)
Supplement: Supplementary file 1 — Supplementary file1 (DOCX 30 KB) [file 41999_2024_1132_MOESM1_ESM.docx]

# Appendix 1 Detailed characteristics of the included papers

| Author | Year | Country | Funding | Aim | Design and data collection method | Sample |
| --- | --- | --- | --- | --- | --- | --- |
| Patients | | | | | | |
| Rush, K. et al. | 2011 | Canada | Grants from the British Columbia Rural and Remote Network and the Health Resource Office, University of British Columbia. | To understand the meaning of weakness for older adults’ and their perceptions of its association with ageing. | Qualitative in-depth interviews, analysed from a social constructivist paradigm. Participants were purposively selected through wellness centres and church groups for maximum variation according to age, place, marital status and living arrangements. Inclusion criteria were age over 65 and able to consent. | 13 participants. All were Caucasian, 10 were women, 10 were in their 80s, 1 in 90s. 6 had multiple health conditions, 7 were widowed, 5 were married/remarried, 5 lived alone, 5 lived with their spouse. 11 had fallen within the past 5 years. |
| Evans C. J. et al. | 2011 | USA | Amgen Inc. (formerly Applied Molecular Genetics Inc.) an American multinational biopharmaceutical company headquartered in Thousand Oaks, California. One of the world's largest independent biotechnology companies. C-F.C., S.G. are employed by Amgen and hold stocks in Amgen. W.J.E. and W.D. received funds for recruitment of study participants. W.D. received a grant for data collection from Amgen. D.L.P. received honorarias for his consulting services from Amgen. C.J.E., K.A.F., W.J.E., B.R.F., W.D., L.P.F., B.D-S., and D.L.P. provided consulting services to Amgen. | To develop a patient-reported outcome (PRO) to assess reduced muscle strength in sarcopenia | Qualitative interview study using grounded theory analysis. Participants were recruited by investigator (W.J.E.) at the University of Arkansas for Medical Sciences (UAMS) and interviewed using a semi-structured concept-elicitation guide. Face-to-face meeting of authors to decide on PRO measures for inclusion. Followed by a further 12 cognitive debriefing interviews. | 12 participants. 9 females and 3 males ranging from 64 to 89 years old. 75% were white and 66.7% had attended some college or had a college or graduate degree. Other inclusion criteria; gait speed < 0.9 metres/second and BMI≤30 kg/m^2^. 66.7% of participants scored between 8.00 and 9.00 from a best score of 12 on the Short Physical Performance Battery assessment. Participants with any condition that could cause secondary sarcopenia were excluded (e.g. severe COPD). |
| Beaudart. C. et al. | 2020 | Belgium, Spain | This work was supported by the European Society for Clinical and Economic Aspects of Osteoporosis, Osteoarthritis and Musculoskeletal Diseases (ESCEO). OB, JYR, and CB are shareholders in SarQoL sprl. | To identify critical outcomes for sarcopenia and to select the 5 most important outcomes that will be used in a further discrete-choice experiment (DCE). | A literature review followed by an expert panel and 3 focus groups with sarcopenic participants. Focus group participants were “recruited in Belgium and Spain” but with no more detail. | 19 “sarcopenic patients”, 6 men, 13 women, mean age 78. Further details including the method of diagnosing sarcopenia are not described. |
| Hiligsmann, M. et al. | 2020 | Belgium, France, Germany, Italy, Spain, and Switzerland | European Society for Clinical and Economic Aspects of Osteoporosis, Osteoarthritis and Musculoskeletal Diseases (ESCEO). CB, OB, and JYR are shareholders of SarQoL sprl, a spin-off of the University of Liège. | To evaluate patient's priorities for sarcopenia outcomes (from five pre-determined outcomes decided by prior work by Beaudart et al. 2020). | Discrete choice experiment by paper survey carried out at home or in clinic. | 216 participants (46 from Belgium, 30 from France, 18 from Germany, 50 from Italy, 39 from Spain, and 33 from Switzerland). The respondents had a mean age of 77.9 years, and 68% were female. 44 (21%) had received college or university education. Race/ethnicity of participants was not reported. Sarcopenia was diagnosed according to European working group on sarcopenia in older people (EWGSOP), foundation for the national institutes for health (FNIH), or International working group on sarcopenia (IWGS) definitions. |
| Zanker, J. et al | 2022 | Australia | Australian Government Research Training Program (TRP) Scholarship.; Australian Medical REsearch Future Fund; Dairy Australia, California Dairy Research Foundation, National Dairy Council, Aarhus University Hospital and Danish Dairy Research Foundation, Fonterra Co-operative Group Ltd, Dutch Dairy Association, Dairy Council of California, Dairy Farmers of Canada, the Centre national interprofessionnel de l'economie laitiere, University of Melbourne, Austin Hospital Medical Research Foundation and Sir Edward Dunlop Medical Research Foundation.; Deakin University, Amgen, Department of Health and Human Services (DHHS), and the Norman Beischer Foundation.; National Health and Medical Research Council; National Heart Foundation Future Leader Fellowship (ID: 102817).; NHMRC CRE 1102208 and Hospital Research Foundation.; NHMRC Postgraduate Scholarship, grant number 2003179.; NHMRC project grant (APP1099173).; Royal Perth Hospital Career Advancement Fellowship (CAF 130/2020) | To develop guidelines, informed by healthcare consumer values and preferences, for sarcopenia prevention, assessment, and management for use by clinicians and researchers in Australia and New Zealand. | Three-phase modified Consumer Expert Delphi study. Email invitations were sent to consumer groups across Australia and New Zealand, and Task Force members (an expert panel of healthcare professionals and academics) used existing networks and social media to invite consumer participants. | Consumers participating in Phase 2 (n= 24), had mean±SD age of 67.5±12.8years and 16 (67%) were women. The majority (n= 10, 42%) described themselves as ‘interested in sarcopenia’, followed by ‘consumer of healthcare’, (n= 7, 29%). Six (25%) people were ‘living with sarcopenia’ or ‘believed they had sarcopenia’ and one (4%) described themselves as a ‘carer’. n=23 (96%) were Caucasian. Most were a "self-funded retiree" n=11 (46%) with n=6 (25%) receiving government support pension. |
| Health and care professionals | | | | | | |
| Yaxley, A. et al. | 2011 | Australia | Flinders University and the Department of Rehabilitation & Aged Care at Repatriation General Hospital for the provision of a research stipend. | To determine whether dietitians understand and use the terms starvation, sarcopenia, and cachexia and provide targeted treatment strategies accordingly. | Cross-sectional survey of members of the Dieticians Association of Australia (DAA). Participants were recruited via a link in a weekly email to the DAA distribution list. | 221 DAA members, including ten student members, accessed the web-based survey (a response rate of approximately 5.5%) 169 participants completed the survey. n=215 (97.3%) respondents were female. Around half worked in metropolitan/urban settings and half in public hospitals. |
| ter Beek, L. | 2016 | Belgium, the Netherlands Norway and Sweden | No funding is mentioned but there is a statement declaring no conflicts of interest. | To determine whether dietitians in selected European countries have ‘sufficient knowledge’ of malnutrition, starvation, cachexia and sarcopenia, and use these terms in their daily clinical work. | Cross-sectional online survey of dieticians recruited via regional and national associations of dieticians. The authors used the same survey as was devised and used by Yaxley A. et al. (2011) | 369 respondents included in analysis, representing a 5.1% response rate. 211 (57.2%) worked in the hospital setting, 17 (4.6%) in primary care, 48 (13.1%) in “municipality” including home care and elderly care. |
| Reijnierse E. M. et al. | 2017 | the Netherlands | European Union’s Horizon 2020 research program PreventIT and the Marie Curie, Sklodowska, Innovative Training Network PANINI both awarded to the last author A.B.M. | To assess knowledge and practice of sarcopenia diagnosis and management among Dutch healthcare professionals. | Longitudinal survey questionnaire study. Participants completed a questionnaire before, directly after, and five months after attending a "Sarcopenia Roadshow" consisting of a 1.5 hrs plenary lecture followed by three parallel workshops. Participants were recruited from four lecture locations spread over the Netherlands (Hertogenbosch, Haarlem, Dordrecht, Texel) between February 2015 and September 2015. | 223 healthcare professionals. 69 (30.9%) were physicians, 32 (14.3%) nurses, 22 (9.9%) GP assistants, 83 (37.2%) physiotherapists and 17 (7.6%) were dietitians. 101 (45.3%) worked in primary care, 51 (22.9%) in nursing homes and 71 (31.8%) in hospitals. Other demographics of participants were not reported. |
| Nakahara S. et al. | 2018 | Japan | None, no conflicts of interest | To evaluate and compare the relative use of sarcopenia and cachexia evaluations among dietitians and associated healthcare professionals in a diverse range of settings. | Online questionnaire survey analysed quantitatively and published on the Facebook group webpage of the Japanese Association of Rehabilitation Nutrition. | 683 HCPs. 178 (26.1%) dietitians  191 (28.0%) physiotherapists  40 (5.9%) occupational therapists  107 (15.7%) speech-language hearing therapists, and  53 nurses (7.8%). |
| Offord, N.J. et al. | 2019 | the United Kingdom | None, no conflicts of interest | To survey UK healthcare professionals to understand how sarcopenia and frailty are diagnosed and managed in current UK practice. | A series of four online questionnaires, the first about sarcopenia and others about frailty in different clinical contexts. The questionnaire was circulated to members of the British Geriatrics Society Sarcopenia and Frailty Research Special Interest Group (SiG) through British Geriatrics Society (BGS) media channels. | 61 HCPs completed the sarcopenia questionnaire (22 consultant geriatricians, 2 general practitioners (GPs), 3 trainee geriatricians, 7 specialist nurses, 13 Allied Health Professionals (AHPs) and 14 others), representing 49 organisations from a total of 177 hospital-based NHS organisations or health boards in the UK (28%). |
| Kiss N. et al. | 2020 | Australia | None, no conflicts of interest | To determine the awareness, perceptions, and practices of Australian oncology clinicians regarding malnutrition and sarcopenia in people with cancer. | A national cross-sectional survey of Australian cancer clinicians distributed through the membership of professional cancer organizations (Clinical Oncology Society of Australia, Cancer Council Victoria Clinical Network), via the social media platform Twitter and to the professional networks of the research team via email invitation. | 111 participants. Dieticians n=42 (38%), nurses n=38 (34%), medical practitioner n=16 (14%) and physiotherapist n=5 (5%) exercise physiologist n=2 (2%) other allied health n=8 (7%). n= 90 (81%) worked in public hospitals and n=85 (76%) worked in metropolitan areas. The gender and ethnicity of survey respondents was not reported. |
| Silva, R.F. et al. | 2020 | Brazil | Not mentioned | To describe the knowledge and practices of primary care nurses on sarcopenia screening. | Qualitative interviews. Semi-structured interviews analysed using thematic analysis. Nurses were interviewed in their place of work, interviews lasted 20-30 mins. | 24 nurses. Participants were mostly female (n = 21), aged between 23 and 44 years. 23 interviewees had never participated in gerontology training. |
| Yeung, S. S. Y. et al. | 2020 | Australia and New Zealand | The European Union’s Horizon 2020 research and innovation programme. | To describe the current knowledge and practice of sarcopenia diagnosis and treatment among health‐care professionals before, directly after and 6 months after a professional development event on sarcopenia. | Longitudinal survey study. Participants completed questionnaires before, after, and at 6 months following a professional development event termed "the sarcopenia roadshow". This was a 2 hour long didactic teaching delivered by a dietician and geriatrician. | 250 healthcare professionals. 147 (59.8%) dieticians, 54 (22%) physicians, 36 (14.6%) nursing, 9 (3.7%) others. 202 (83%) of participants were female. 132 (54.5%) worked in the hospital setting, 65 (26.9%) in primary care and 36 (14.9%) in community services. |
| Guralnik J. M. et al. | 2022 | USA | Aging in Motion Coalition (A project of the Alliance for Aging Research) Abbott Nutrition, Biophytis, Cytokinetics, Metabolic Technologies, LLC, Nestle Health Science, and Pfizer,Inc. provided funding to the Alliance for Aging Research. | To evaluate US physicians' familiarity with sarcopenia and its use in their practices. | Qualitative interviews recruited through convenience sampling. An online survey, recruited through email invitation through Medscape. | 9 physicians undertook qualitative interviews lasting 30 mins.  253 full-time physicians board certified in either internal medicine, physical medicine and rehabilitation (PM&R), family medicine or geriatrics. |
| Lu F. et al. | 2023 | China | Grants from the Medical Science and Technology Project of Zhejiang Province and Basic Public Welfare Research Program of Zhejiang Province. | To analyse and compare the knowledge, attitude, and practice regarding sarcopenia between orthopaedics and geriatrics professionals. | Cross-sectional online survey of orthopaedics and geriatrics professionals selected through convenience sampling. Participants were employed in two tertiary hospitals in southeastern China. | 220 professionals. 103 doctors (46.8%) 117 nurses (53.2%). 176 from orthopaedics departments and 44 from geriatric medicine departments. 120 (54.5%) were female. |
| Verstraeten, L. M. G. et al. | 2023 | the Netherlands | A.B.M. reports grants from Danone Nutricia Research during the conduct of the study. J.P.v.W. reports that she is an employee of Danone Nutricia Research. L.M.G.V. and C.G.M.M. declare that they have no conflicts of interest. The collaboration project is co-funded by the PPP Allowance made available by Health∼Holland, Top Sector Life Sciences & Health, and Top Sector Agri & Food to stimulate public-private partnerships and includes an in-cash and in-kind contribution from Danone Nutricia Research. Vrije Universiteit, Amsterdam UMC, and Cordaan made in-kind contribution. | To assess sarcopenia awareness and knowledge; perception of responsibility; current screening, diagnosis, and treatment practices; and barriers and enablers to screening/diagnosis and treatment of sarcopenia among geriatric rehabilitation health care professionals in the Netherlands. Adequate knowledge was assessed against EWGSOP/EWGSOP2 guidelines. | Cross-sectional online survey. Participants were recruited via mail invitation, via newsletter circulation, and through nursing/rehabilitation and allied health professional networks in the Netherlands. | 501 HCPs, (n = 61) 12.2% were physicians, (n = 115) 23.0% PT/OTs, (n = 152) 30.3% dietitians, (n =98) 19.6% nurses, and (n = 55) 11.0% health care assistants. The median age was 42 years [IQR: 30-54] and 87.2% (n = 437) were females. |
| The lay public | | | | | | |
| Van Ancum, J.M. et al. | 2020 | the Netherlands | The European Union’s Horizon 2020 research and innovation programme (Grant: 689238, 675003). | To describe the current knowledge about sarcopenia in a cohort of community-dwelling adults attending health educational events. And to correlate self-perceived muscle health with objective muscle measures. | Cross-sectional survey and measurements, including a sarcopenia awareness questionnaire, bioimpedance analysis, hand-grip strength and 4m walk speed test, height/weight. Participants were recruited at three different hospital/health clinic sites after attending a “sarcopenia awareness show” in the Netherlands. | 197 older people. The median age was 67.9 years (IQR = 57.0-75.1). n=140 (71%) of the participants were female. n=129 (65%) were retired with n=61 (31.3%) having had a career in healthcare. n=138 (70%) reported muscle strengthening activities ≥2 times per week. n=10 (5.3%) had sarcopenia according to the EWGSOP guidelines. Participants had high levels of education, low levels of MLTC and polypharmacy. Race/ethnicity was not reported. |
| Gilliot, S. et al. | 2021 | Belgium | None reported | To investigate with Google Trends whether the clinical importance of sarcopenia is reflected in public interest in the disease. Sarcopenia was compared with search trends on the topics of "dementia" "polypharmacy" "osteoporosis" and "frailty". | Using the search tool Google Trends, a free and publicly accessible tool analysing web queries made via the Google search engine and displaying the results on a  normalised scale. | Google trends was queried for data from Jan 2004 to Jan 2020 with geographical location not limited (worldwide). |
| Keng S. et al. | 2023 | Malaysia | None, no conflicts of interest | To investigate "knowledge of sarcopenia" among the public in Malaysia | Cross-sectional online survey analysed quantitatively and disseminated via social media platforms. | 202 participants. Mean age 49 (+- 12.65), 151 (74.8%) women. 55 (27.2%) Malay, 120 (59.4%) Chinese, 21 (10.4%) Indian. The highest level of education was degree-level for 90 (44.6%). 181 (89.6%) were independent of ADLs. Participants were assumed not to have sarcopenia based on 186 (92.1%) not using a walking aid and reporting no difficulty walking 100m 186 (92.1%). |
|  | 2023 | Taiwan | None, no conflicts of interest | To evaluate the level of awareness of sarcopenia among older adults and to develop and test the reliability and validity of the Sarcopenia Knowledge Questionnaire. | Cross-sectional survey of adults aged 65 years and over recruited through convenience sampling at a hospital outpatient department. Selection criteria were literacy in Mandarin Chinese and an ability to consent to participate. | 293 participants from Northern Taiwan. Mean age 73,12 +-5.95 years, range 66-95 years, 181 (61.8%) women. 215 (73.4%) exercised more than once per week or daily. 27 (9.2%) had >3 comorbidities. |
